# Supplementary material for: Resolving the full spectrum of human genome variation using Linked-Reads
Source: Genome Res. 2019 Apr;29(4):635–45. doi: 10.1101/gr.234443.118 (PMC6442396; doi:10.1101/gr.234443.118)
Supplement: Supplemental Material [file supp_gr.234443.118_Supplemental_longranger-2.2.zip › longranger-2.2.tar.bz2!/lr2.2-public/lib/stan/cmdstan/stan/src/docs/stan-reference/programs/ode/sho/sho-trajectory.pdf]

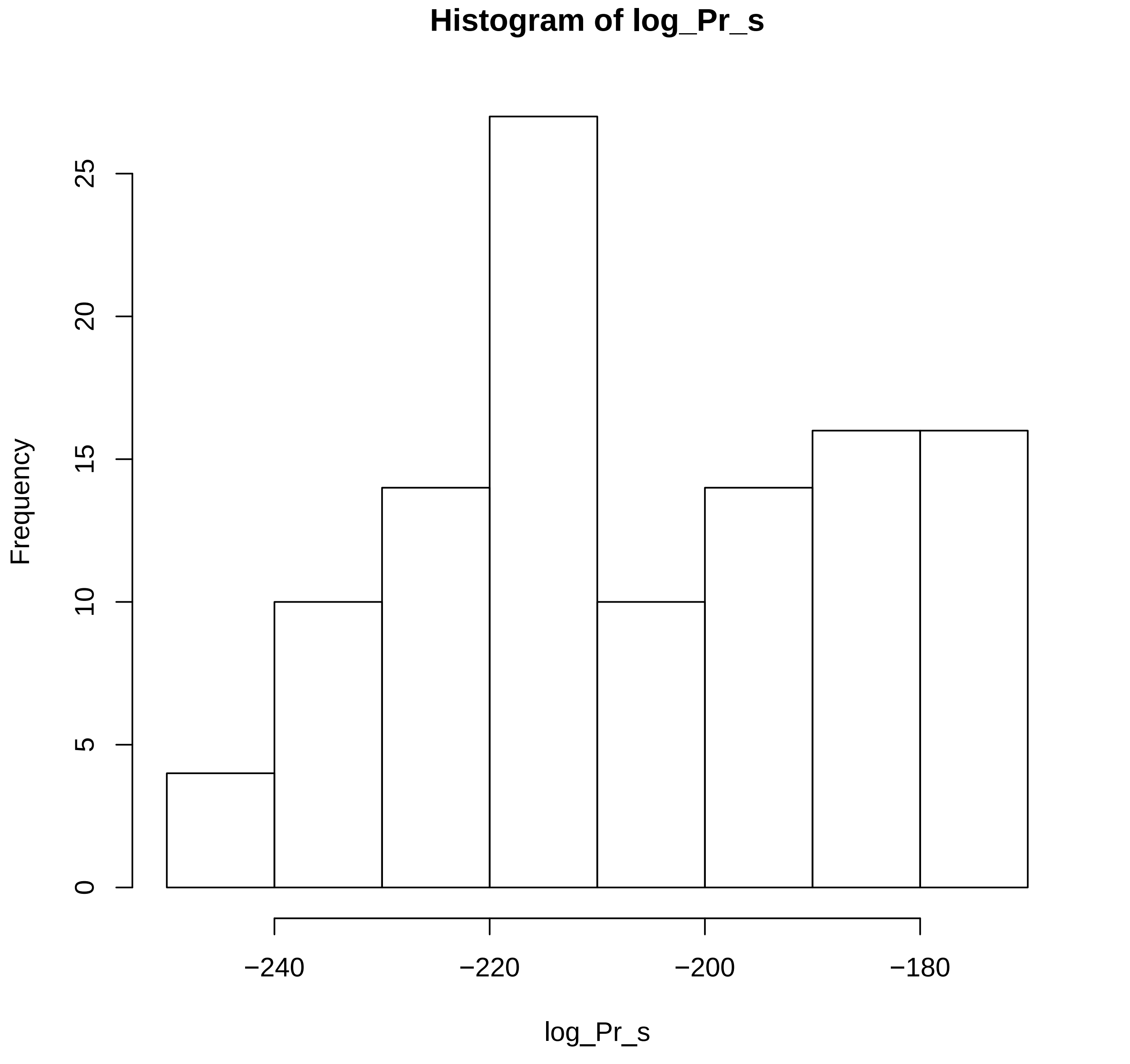
Histogram of log_Pr_s
Frequency
0
5
10
15
20
25
−240
−220
−200
−180
log_Pr_s
